# Beyond the Microbial Hypothesis: Shared Genetic, Osteoimmune and Vascular Mechanisms in the Oral–Brain Axis Linking Periodontitis and Alzheimer's Disease

**DOI:** 10.1111/ejn.70748

**Published:** 2026-11-01

**Authors:** Salma Kabbashi, Mai Ahmed, Manogari Chetty

**Affiliations:** ^1^ Craniofacial Biology, Pathology, and Radiology Department, Faculty of Dentistry University of the Western Cape Cape Town South Africa

**Keywords:** Alzheimer's disease, apolipoprotein E, endocan, OPG/RANKL, periodontitis

## Abstract

Periodontitis, a chronic inflammatory disease of the tooth‐supporting tissues, is increasingly linked to Alzheimer's disease (ad), yet mechanistic accounts of this ‘oral–brain axis’ have been dominated by microbial translocation and the gingipain hypothesis. This review argues that the convergence between periodontitis and ad extends well beyond microbes, integrating shared genetic, osteoimmune and vascular‐inflammatory mechanisms that are rarely considered together. Genetic susceptibility reaches beyond APOE ε4 to the osteoprotegerin (OPG) axis, with APOE ε4 linking the two conditions through impaired amyloid‐β clearance, blood–brain barrier dysfunction and altered lipid handling. The OPG/RANK/RANKL system emerges as a bone–brain (osteoimmune) interface, in which OPG shows a dual‐role paradox, reduced locally in periodontal bone loss yet elevated systematically in ad, while soluble RANKL displays neuroprotective properties. Because OPG acts as a decoy receptor for RANKL, we hypothesise that elevated OPG in ad limits RANKL‐dependent microglial amyloid‐β clearance. Endothelial dysfunction, reflected in circulating endocan, provides a shared vascular readout that is raised in both diseases and responsive to periodontal therapy. We propose that these genetic, microbial and vascular inputs converge on microglial priming and trained immunity, a unifying immunological node through which chronic peripheral inflammation may act during the decades‐long preclinical phase of ad. Current evidence nonetheless remains predominantly associative, causality is unproven and mechanistic data rely heavily on animal models. The oral–brain axis is therefore best regarded as a promising yet still unproven framework in which osteoimmune and vascular pathways, and candidate biomarkers such as endocan, represent underexplored targets for future longitudinal and interventional research.

## Graphical abstract

Periodontitis is linked to Alzheimer's disease (ad) beyond the microbial hypothesis. This review reframes the oral–brain axis as an osteoimmune and vascular problem: The OPG/RANKL system acts as a bone–brain interface in which elevated OPG may limit neuroprotective RANKL, and circulating endocan provides a shared, treatment‐responsive vascular biomarker. Genetic, microbial and vascular inputs converge on microglial priming during the long preclinical phase of ad.

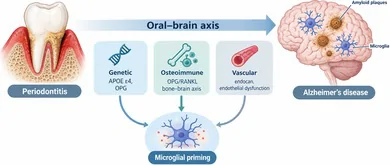

**Abbreviations**
Aβ: amyloid‐β
ad
: Alzheimer's disease
APOE (ε4): apolipoprotein E (ε4 allele)
BBB: blood–brain barrier
CNS: central nervous system
CRP: C‐reactive protein
DALYs: disability‐adjusted life years
ESM‐1: endothelial cell‐specific molecule‐1 (endocan)
GAIN: GingipAIN Inhibitor for the treatment of Alzheimer's disease (trial)
GCF: gingival crevicular fluid
GSK‐3β: glycogen synthase kinase‐3β
IL‐1β: interleukin‐1β
IL‐6: interleukin‐6
LMICs: low‐ and middle‐income countries
LPS: lipopolysaccharide
MCI: mild cognitive impairment
Mfsd2a: major facilitator superfamily domain‐containing protein 2a
MoCA: Montreal Cognitive Assessment
MR: Mendelian randomization
NF‐κB: nuclear factor kappa‐B
NHANES: National Health and Nutrition Examination Survey
OPG: osteoprotegerin
RANK: receptor activator of nuclear factor kappa‐B
RANKL: receptor activator of nuclear factor kappa‐B ligand
RCT: randomised controlled trial
sRANKL: soluble RANKL
TLR4: Toll‐like receptor 4
TNF(‐α): tumour necrosis factor(‐α)
TRAIL: TNF‐related apoptosis‐inducing ligand
WHO: World Health Organization

## 1 Introduction

Periodontitis is a chronic inflammatory disease with serious and irreversible destructive effects that extend beyond the oral cavity (World Health Organization [WHO] 2022). Estimates of its global prevalence vary considerably with case definition and surveillance methodology; severe periodontitis is estimated to affect approximately 19% of the adult population, with the burden concentrated in, though not confined to, low‐ and middle‐income settings (WHO 2022). This variability is thought to reflect diagnostic heterogeneity, underreporting and limited surveillance infrastructure (WHO 2022; Papapanou et al. 2018).

Spontaneous teeth loss, bleeding gums, mild discomfort or pain and overall reduction in quality of life are some of the common signs and symptoms of this progressive disease (Papapanou et al. 2018). Periodontitis arises from a complex interaction between dental plaque microbiome, the host immune‐inflammatory response and environmental factors in susceptible people (Zhang et al. 2019). When dysregulated, this immune‐inflammatory response, in addition to driving local tissue destruction in the periodontium, can also disseminate into the systemic circulation. The resulting chronic, low‐grade systemic inflammation has been implicated in the pathogenesis of various extra‐oral conditions (Kinane et al. 2017). A growing body of evidence connects periodontitis to systemic conditions such as cardiovascular disease, diabetes, cancer and Alzheimer's disease (ad), with the associated systemic inflammatory burden particularly implicated in cognitive impairment and neuroinflammation (Isola et al. 2023).

ad is a progressive neurodegenerative disorder that severely impairs memory and cognitive function. It accounts for up to 70% of dementia cases globally and represents a significant public health burden, with care costs exceeding a trillion US dollars annually (Panzarella et al. 2022; Wong 2020). ad ranks as the fourth leading cause of disability‐adjusted life years (DALYs) among individuals aged 75 and older (Zheng et al. 2024). The WHO projects that by 2050, dementia cases will reach around 139 million, with over 60% occurring in low‐ and middle‐income countries (LMICs), placing additional strain on already limited healthcare systems (Shin 2022).

While only 5%–10% of ad cases are familial with Mendelian inheritance (Bekris et al. 2010), the majority are sporadic and linked to aging. Besides nonmodifiable factors like genetics, age, sex and race, modifiable risks such as poor oral hygiene, hypertension, diabetes, depression, inactivity, poor sleep, low education and limited social interaction also contribute to disease development (Evans‐Lacko et al. 2024; Mfene and Pillay 2024). Although the exact aetiology of ad remains unclear, accumulating evidence suggests that brain inflammation plays a central role in disrupting synaptic function, triggering neurodegenerative changes and ultimately leading to cognitive decline (Cichońska et al. 2024). Despite decades of research, the mechanisms driving ad onset and progression are still not fully understood (Scheltens et al. 2021), prompting growing interest in identifying modifiable risk factors and early preventive strategies. Both ad and periodontitis are complex, multifactorial diseases with high global prevalence and significant health burdens. Importantly, ad is now recognised as a condition that begins decades before the onset of clinical symptoms such as mild cognitive impairment (MCI), highlighting the need to investigate early contributing factors (Le Page et al. 2017).

Emerging research links periodontitis to ad, implicating chronic oral inflammation and systemic immune responses in the acceleration of neurodegenerative processes (Kamer et al. 2020; Zhang et al. 2025). Although microbial translocation and the gingipain hypothesis have dominated mechanistic accounts of the oral–brain axis, the convergence between periodontitis and ad extends across several less frequently connected domains. Shared genetic susceptibility reaches beyond APOE4 to the osteoprotegerin (OPG) axis; the OPG/RANK/RANKL system links bone and brain through common inflammatory signalling; and endothelial dysfunction, reflected in circulating endocan, provides a shared vascular readout. These pathways plausibly converge on microglial priming, a common node at which genetic, microbial and vascular influences may be integrated.

## 2 Shared Genetic Susceptibility

### 2.1 APOE ε4: A Converging Risk Factor

Apolipoprotein E (APOE), particularly the ε4 allele (APOE4), is the strongest genetic risk factor for sporadic ad. APOE4 contributes to impaired amyloid‐β (Aβ) clearance and increased Aβ aggregation and is associated with tau hyperphosphorylation and enhanced neuroinflammatory responses (Chai et al. 2021). Furthermore, APOE4 has been associated with blood–brain barrier (BBB) dysfunction and cerebrovascular instability, potentially facilitating the passage of periodontal‐derived inflammatory mediators, microorganisms and bacterial products into the central nervous system (Montagne et al. 2020; Tai et al. 2016). At the cellular level, APOE4 also reprogrammes microglial lipid metabolism and promotes a pro‐inflammatory microglial phenotype, amplifying neuroinflammation and impairing Aβ clearance (Chen et al. 2025; Ebright et al. 2022; Faraji et al. 2024).

Beyond its role in ad, emerging evidence implicates *APOE* in periodontal disease pathogenesis. Gingipains produced by *Porphyromonas gingivalis* can degrade ApoE, thereby disrupting lipid homeostasis and immune regulation (Lönn et al. 2018). *APOE*‐deficient animal models demonstrate hyperlipidaemia and increased susceptibility to periodontal pathogens, suggesting that lipid dysregulation may potentiate host inflammatory responses (Lei et al. 2013; Vasquez et al. 2012). Consistent with this, oral *P. gingivalis* infection produces extensive oxidative damage in ApoE‐knockout mice, directly linking APOE status to the periodontal inflammatory response (Heneka et al. 2025). Additionally, the APOE rs429358 polymorphism, defining the ε4 allele, has been associated with aggressive periodontitis in certain populations (Li et al. 2020), although findings remain population dependent (Borilova Linhartova et al. 2015; Kamer et al. 2015). Furthermore, chronic periodontal inflammation may itself alter systemic ApoE handling, suggesting a bidirectional relationship in which APOE status and periodontal disease reinforce one another (Lönn et al. 2018).

Direct human evidence, although still limited, has begun to connect these observations. In patients with ad, gingival crevicular fluid shows elevated ApoE alongside higher *P. gingivalis* load and pro‐inflammatory mediators, both correlating inversely with cognitive Montreal Cognitive Assessment (MoCA) scores, and crevicular ApoE distinguished periodontitis with and without ad with sufficient accuracy, suggesting it as an accessible biomarker (Sansores‐España et al. 2022). Some data indicate that periodontal status correlates with cognitive impairment specifically in APOE4 carriers (Arévalo‐Caro et al. 2025); however, this interaction is inconsistent, as a large National Health and Nutrition Examination Survey (NHANES III) analysis found that APOE4 did not affect the association between antibodies to periodontal microorganisms and cognition (Merchant et al. 2025).

Mechanistically, APOE4 may serve as a bridge between periodontitis and ad through three interrelated processes: dysregulated lipid metabolism, amplification of inflammatory signalling and increased susceptibility to microbial challenge. This convergence supports the hypothesis that APOE4 carriers with chronic periodontal inflammation may exhibit heightened vulnerability to neurodegenerative processes.

These findings position APOE4 as a central immunometabolic regulator linking peripheral inflammation in periodontitis to neurodegenerative cascades in ad. Crucially, these lipid‐ and inflammation‐related mechanisms ultimately converge on the neuroinflammatory and microglial responses that also lie downstream of the OPG/RANK/RANKL axis, a bone‐regulatory system with an increasingly recognised role in the brain (Chen et al. 2025; Glasnović et al. 2020).

### 2.2 OPG/RANK/RANKL Axis: Beyond Bone Biology

#### 2.2.1 OPG in Periodontitis

OPG is a soluble decoy receptor that binds receptor activator of nuclear factor kappa‐B ligand (RANKL), thereby inhibiting osteoclast differentiation and bone resorption. A balanced RANKL/OPG ratio is essential for maintaining alveolar bone integrity. In periodontitis, reduced OPG levels accompanied by increased RANKL expression promote osteoclastogenesis and progressive alveolar bone destruction (Asif et al. 2022; Giannopoulou et al. 2012). Genetically, the OPG +1181 [G/C] polymorphism (rs2073618) results in a nonsynonymous amino acid substitution within the signal peptide region, potentially affecting protein secretion efficiency (Nakajima et al. 1999; Zhao et al. 2005). Population‐based studies have reported inconsistent associations; however, recent data suggest that the CC genotype may increase susceptibility to periodontitis in specific populations (Kabbashi et al. 2026).

#### 2.2.2 OPG in AD

Beyond its role in skeletal biology, OPG has been implicated in neuroinflammatory and vascular mechanisms associated with ad. Elevated plasma OPG levels have been reported in ad patients and are associated with reduced total brain volume and markers of vascular dysfunction (Guo et al. 2025; Jefferson et al. 2007). OPG also interacts with TNF‐related apoptosis‐inducing ligand (TRAIL), thereby influencing immune regulation and apoptotic signalling within the central nervous system (Freeman et al. 2024).

This vascular association aligns with a broader role for endothelial dysfunction in ad. Endothelial activation and increased BBB permeability, which may precede amyloid deposition, are driven in part by NF‐κB‐associated inflammatory endothelial gene modules that promote endothelial reprogramming, junctional disruption, immune‐cell infiltration and propagation of neuroinflammatory responses (Brito 2019; McLarnon 2021; Pinals et al. 2025; Tarawneh 2023; Yamazaki and Kanekiyo 2017; Yue et al. 2024). Positioned within this framework, the elevated OPG observed in ad may represent a maladaptive facet of endothelial‐inflammatory activation rather than a purely compensatory response, a biologically plausible interpretation that remains to be tested directly.

Interestingly, while reduced OPG contributes to bone destruction in periodontitis, elevated systemic OPG levels in ad may reflect a context‐dependent response to persistent inflammation, highlighting the dual‐role paradox of OPG in bone versus brain pathology. This paradox is increasingly interpreted within a bidirectional bone–brain (osteoimmune) axis, in which RANKL/OPG signalling and shared inflammatory and Wnt/β‐catenin pathways couple skeletal and cerebral pathology (Liu, Liu, et al. 2024; Xu et al. 2026). Consistent with this framework, osteoporosis and ad frequently co‐occur: Reduced bone mineral density is associated with roughly a twofold higher risk of ad and dementia, the relationship appears bidirectional and the APOE4 variant is itself associated with osteoporosis (Nasme et al. 2025) Because OPG acts as a decoy receptor for RANKL, elevated OPG in ad may not only signal vascular‐inflammatory stress but also limit the availability of neuroprotective RANKL, offering a unifying explanation for the opposing directions of OPG and RANKL observed in the brain.

### 2.3 RANKL as a Neuroprotective Modulator

Recent Mendelian randomization analyses demonstrate a significant inverse association between circulating soluble RANKL (sRANKL) and ad risk (Guo et al. 2025). Mechanistically, RANKL enhances lysosomal acidification in microglia via upregulation of vacuolar H^+^‐ATPase, thereby promoting Aβ clearance (Lee and Nixon 2022; Serrano et al. 2009).

It has been proposed that sRANKL may cross the BBB and interact with neural RANK receptors, whereas OPG is less likely to do so owing to its larger molecular size; however, direct experimental evidence of RANKL transport across the human BBB remains limited, and this mechanism requires confirmation (Guo et al. 2025; Guerrini et al. 2015). Clinical observations that denosumab (a monoclonal antibody against RANKL) have been associated with cognitive side effects further support the functional relevance of RANKL signalling in the central nervous system (Oteo‐Álvaro et al. 2023).

In aggregate, the RANKL/RANK/OPG axis represents a key regulatory interface between peripheral inflammation and central immune responses. Dysregulation of this pathway may simultaneously drive osteoclastogenesis in periodontal tissues and alter microglial activation states, supporting a plausible mechanism of bone–brain cross‐talk (Glasnović et al. 2020; Kong et al. 2025; Patil and Desai 2014). Whereas the genetic and molecular mechanisms above define host susceptibility, the periodontal microbiota supplies the exogenous trigger that engages these pathways.

## 3 Microbial Contribution to the Oral–Brain Axis

Periodontitis is characterised by dysbiosis of the oral microbiome, enrichment of pathobionts alongside depletion of health‐associated commensals. This dysbiotic shift, involving organisms such as *P. gingivalis* , *Tannerella forsythia* and *Fusobacterium nucleatum* with parallel loss of commensals including *Streptococcus*, *Rothia* and *Neisseria*, has been consistently associated with ad and MCI, and its magnitude appears to track the severity of cognitive decline (Chaple‐Gil et al. 2025). Among these organisms, *P. gingivalis* has been the most extensively studied.

### 3.1 *P. gingivalis*

Chronic infection with *P. gingivalis* has been detected in ad brain tissue, with gingipains identified in neurons and glial cells (Dominy et al. 2019). Gingipains promote microglial activation, induce pro‐inflammatory cytokine release, increase BBB permeability and stimulate Aβ production (Ilievski et al. 2018; Poole et al. 2013). Inhibition of gingipains in animal models reduces bacterial load and attenuates neurodegeneration, supporting a potential causal role for *P. gingivalis* in ad pathogenesis (Dominy et al. 2019).

Translation to humans, however, has been more equivocal. In the Phase 2/3 GingipAIN Inhibitor for the treatment of Alzheimer's disease (GAIN) trial, the gingipain inhibitor atuzaginstat did not meet its coprimary cognitive and functional endpoints in the overall cohort of patients with mild‐to‐moderate ad; only a prespecified subgroup with detectable salivary *P. gingivalis* showed dose‐dependent slowing of cognitive (but not functional) decline, leaving the causal contribution of *P. gingivalis* supported but unconfirmed (Sabbagh and Decourt 2022).

Additional mechanisms include *P. gingivalis* lipopolysaccharide activating TLR4/NF‐κB signalling (Zhang et al. 2018), increased BBB permeability via the Mfsd2a/Caveolin‐1 transcytosis pathway (Lei et al. 2023) and bacterial extracellular vesicles that cross the BBB to trigger neuroimmune responses (Ha et al. 2023). *P. gingivalis* oral infection also activates the classical complement pathway and elicits brain TNF, IL‐6 and IL‐1β, further amplifying neuroinflammation (Heneka et al. 2025).

### 3.2 *F. nucleatum*

Accumulating evidence indicates that *F. nucleatum* may exacerbate ad pathology. In transgenic ad mouse models, oral administration of *F. nucleatum* resulted in worsened cognitive performance, increased amyloid plaque deposition, tau hyperphosphorylation and enhanced microglial activation (D. T. Wu et al. 2023; H. Wu et al. 2022).

Intriguingly, *F. nucleatum* has been identified among the most abundant genera in a cohort of periodontitis patients (Kabbashi et al. 2025), consistent with its proposed role in neuroinflammatory susceptibility.

### 3.3 Mechanisms of Neuroinvasion

Periodontal pathogens and their products are thought to reach the central nervous system by two principal routes: hematogenous dissemination, favoured by the transient bacteraemia, systemic inflammation and BBB compromise that accompany periodontitis (Singhrao et al. 2016); and retrograde spread along peripheral nerves, such as the trigeminal and olfactory pathways (Ha et al. 2023). Once within the brain, microbial components interact with resident immune cells, and molecular mimicry, whereby bacterial antigens resemble host proteins, has been proposed as an additional trigger of autoimmune‐like neurodegenerative responses (Singhrao and Olsen 2019).

Beyond direct invasion, systemic pro‐inflammatory cytokines released during chronic periodontitis, notably IL‐6 and TNF‐α, may sustain microglial activation and drive downstream Aβ deposition and tau pathology, providing an indirect route by which peripheral inflammation is transmitted to the brain (Abbayya et al. 2015; Hashim et al. 2024; Patel et al. 2025).

These pathways are increasingly corroborated by neuroimaging: Structural and functional studies link severe periodontitis to reduced cortical thickness (Rubinstein et al. 2024), altered functional connectivity (Ye et al. 2025) and increased Aβ retention in regions vulnerable to ad (Gil‐Montoya et al. 2025), indicating that microbial dissemination and sustained systemic inflammation may translate into measurable neuroanatomical and functional change.

## 4 Endothelial Dysfunction and the Vascular‐Inflammatory Axis

### 4.1 Endocan as a Shared Biomarker

Endocan (ESM‐1) is an endothelial‐derived dermatan sulphate proteoglycan released by activated vascular endothelium in response to pro‐inflammatory cytokines and proangiogenic signals; its circulating concentration therefore serves as a readout of endothelial activation and microvascular inflammation, reflecting its roles in vascular permeability, leukocyte migration and inflammatory signalling (Liu, Bai, and Feng 2024). Elevated serum endocan has been reported in ad and correlates inversely with cognitive performance (Varan et al. 2018), while raised gingival crevicular fluid levels in chronic periodontitis track clinical markers of periodontal inflammation (Tayman et al. 2020) and fall significantly after non‐surgical periodontal therapy (Karci and Sokmen 2025). Because it is elevated in both conditions, correlates inversely with cognition and responds to treatment, endocan is an attractive single, accessible marker of the shared vascular‐inflammatory burden linking periodontitis and ad and potentially of that burden's response to therapy.

Endothelial dysfunction is increasingly recognised as an early event in ad pathogenesis, characterised by oxidative stress, nitric oxide imbalance and activation of inflammatory endothelial pathways (Montagne et al. 2020; Custodia et al. 2023; Nation et al. 2019; Sweeney et al. 2018). Vascular and endothelial impairment may even precede amyloid deposition, indicating that microvascular injury could act as an initiating factor in ad rather than a secondary consequence of neurodegeneration (Tarawneh 2023; de Vries 2022). APOE4 carriers show heightened vulnerability to inflammation‐induced cerebrovascular impairment, reinforcing the convergence of genetic susceptibility and vascular dysfunction within the oral–brain axis (Kapoor et al. 2025). Endocan thus offers a candidate readout of this shared endothelial‐inflammatory pathway.

### 4.2 Neuroimmune Priming and Microglial Activation

A recurring theme across the genetic, microbial and vascular mechanisms described above is the priming of microglia, the resident immune cells of the brain. Microglial priming refers to a state in which prior or ongoing peripheral immune stimulation shifts microglia towards a sensitised phenotype, such that subsequent inflammatory signals provoke an exaggerated neuroinflammatory response (Heneka et al. 2015). Chronic periodontitis offers a plausible source of such repeated peripheral stimulation through sustained low‐grade endotoxaemia, circulating pro‐inflammatory cytokines and intermittent bacteraemia.

Beyond acute priming, sustained peripheral inflammation can epigenetically reprogramme microglia into a state of long‐lasting innate immune memory (trained immunity). Epidemiological data indicate that inflammatory insults up to two decades earlier increase dementia risk, a timeframe consistent with chronic periodontitis acting during the long preclinical phase of ad (Heneka et al. 2015).

Peripheral inflammatory mediators and microbial components associated with periodontitis, including lipopolysaccharide, IL‐6 and TNF‐α, can signal to the brain through humoral, neural and cellular routes, converging on microglial activation (Dominy et al. 2019; Heneka et al. 2015). Once primed, microglia adopt a transcriptional and metabolic profile, described in disease‐associated microglia, that is characterised by altered lipid handling, lysosomal stress and heightened cytokine output (Deczkowska et al. 2018). This state connects directly to the APOE4‐driven microglial lipid dysregulation and the OPG/RANKL‐associated immune signalling discussed in the preceding sections, suggesting that microglial priming may act as a common downstream node integrating genetic susceptibility, microbial exposure and vascular dysfunction (Figure 1).

**FIGURE 1:**
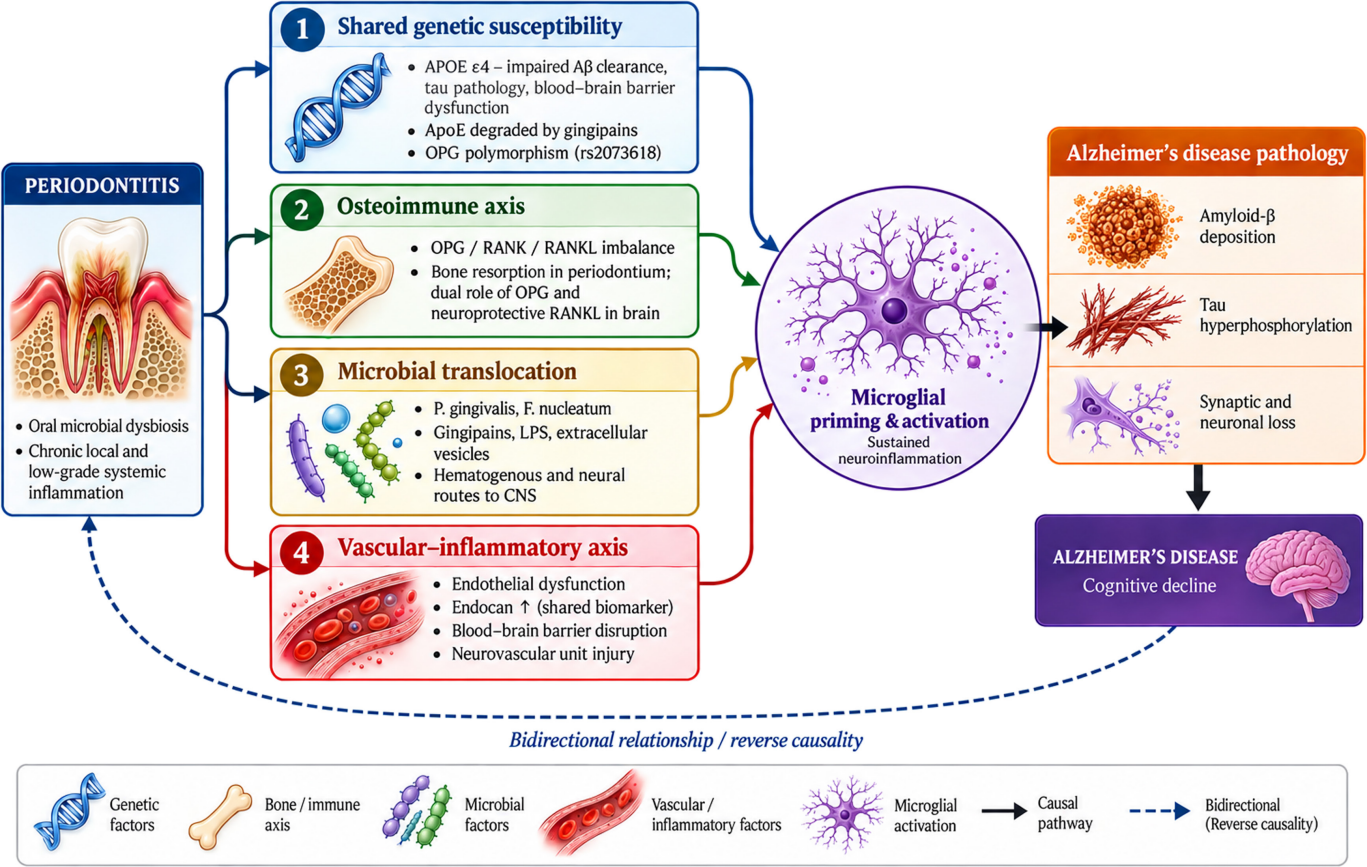
Convergence of shared periodontitis–Alzheimer's mechanisms on microglial priming.

It should be emphasised that microglial priming remains a mechanistic hypothesis in the context of the oral–brain axis. Much of the supporting evidence derives from animal models and in vitro systems, and the extent to which periodontitis‐driven priming translates into clinically meaningful neurodegeneration in humans remains under investigation (Table 1).

**TABLE 1:** Shared mechanisms linking periodontitis and Alzheimer's disease across the genetic, osteoimmune, microbial, vascular and environmental axes.

Mechanism/axis	Role in periodontitis	Role in Alzheimer's disease	Direction and evidence	Key references
Shared genetic susceptibility (APOE ε4, OPG)	APOE ε4/rs429358 associated with aggressive periodontitis (population dependent); OPG +1181 rs2073618 (CC) associated with periodontitis susceptibility	APOE ε4 = strongest genetic risk for sporadic ad (impaired Aβ clearance, tau, neuroinflammation and BBB dysfunction); no established OPG genetic‐variant association with ad	APOE ε4 strong in ad; periodontitis associations inconsistent/population dependent	Chai et al. (2021); Montagne et al. (2020); Li et al. (2020); Nakajima et al. (1999); Kabbashi et al. (2026)
OPG/RANK/RANKL signalling (osteoimmune axis)	↓ OPG with ↑ RANKL drives osteoclastogenesis and alveolar bone loss	↑ plasma OPG (maladaptive vascular‐inflammatory; ↓ brain volume); ↓ soluble RANKL linked to higher ad risk; RANKL neuroprotective (Aβ clearance, ↓ GSK‐3β/tau); OPG‐TRAIL modulates CNS apoptosis	Dual‐role paradox (OPG ↓ in bone/↑ systemically in ad); osteoporosis–ad comorbidity; emerging (MR + observational)	Asif et al. (2022); Jefferson et al. (2007); Guo et al. (2025); Freeman et al. (2024); Glasnović et al. (2020); Nasme et al. (2025)
Periodontal microbiota	Keystone pathogens ( *P. gingivalis* and *F. nucleatum* ) drive dysbiosis and periodontal tissue destruction	Detected in ad brain; gingipains and LPS (TLR4/NF‐κB) → microglial activation, BBB permeability and Aβ; *F. nucleatum* worsens pathology in models	Strong mechanistic (animal/in vitro); human causal link unconfirmed (GAIN RCT negative overall)	Dominy et al. (2019); Zhang et al. (2018); D. T. Wu et al. (2023); H. Wu et al. (2022); Sabbagh and Decourt (2022)
Endothelial/vascular (endocan)	↑ GCF endocan; levels fall after non‐surgical periodontal therapy	↑ serum endocan, inversely correlated with cognition; endothelial dysfunction may precede amyloid deposition	↑ in both conditions; treatment responsive; candidate shared biomarker (emerging)	Varan et al. (2018); Tayman et al. (2020); Karci and Sokmen (2025)
Systemic inflammation → microglial priming (convergence node)	Chronic low‐grade systemic inflammation (↑ CRP, IL‐6 and TNF‐α)	Primes and sustains microglial activation → neuroinflammation, Aβ deposition and tau pathology	Proposed convergence node (hypothesis)	Heneka et al. (2015); Deczkowska et al. (2018)
Shared environmental and lifestyle risk factors	Aging, smoking, diabetes, poor oral hygiene, poor nutrition and socio‐economic disadvantage	Same factors are established modifiable dementia risk factors, acting largely through chronic systemic inflammation	Shared and modifiable; periodontal therapy lowers inflammatory markers and may benefit cognition (mixed RCT/cohort)	Papapanou et al. (2018); Evans‐Lacko et al. (2024)

*Note:* Arrows denote increased (↑) or decreased (↓) levels.

Abbreviations: Aβ, amyloid‐β; BBB, blood–brain barrier; GCF, gingival crevicular fluid; GSK‐3β, glycogen synthase kinase‐3β; LPS, lipopolysaccharide; MR, Mendelian randomization; OPG, osteoprotegerin; RANKL, receptor activator of nuclear factor kappa‐B ligand; RCT, randomised controlled trial; TRAIL, TNF‐related apoptosis‐inducing ligand.

## 5 Shared Environmental and Lifestyle Risk Factors

Several modifiable and nonmodifiable factors independently increase the risk of both periodontitis and ad, and their shared effect appears to be mediated largely through chronic systemic inflammation. Advancing age is the strongest risk factor for both conditions, reflecting immunosenescence and ‘inflammaging’, a progressive, low‐grade rise in pro‐inflammatory mediators that predisposes to both periodontal tissue breakdown and neurodegeneration (Kinane et al. 2017; Heneka et al. 2025). Smoking is a major modifiable driver of periodontitis through impaired periodontal healing and microbial dysbiosis (Kinane et al. 2017) and is likewise an established modifiable risk factor for dementia, acting via oxidative stress, vascular injury and heightened systemic inflammation (Livingston et al. 2024). Type 2 diabetes shares a well‐recognised bidirectional relationship with periodontitis, each condition worsening the other (Kinane et al. 2017), and is also a modifiable risk factor for dementia (Livingston et al. 2024). Poor oral hygiene is a proximal cause of periodontitis (Kinane et al. 2017), and the resulting tooth loss has been independently associated, in dose‐dependent fashion, with increased risk of cognitive impairment and dementia (Fang et al. 2018). Nutritional factors also contribute: dietary patterns of high inflammatory potential are linked to worse outcomes, whereas anti‐inflammatory patterns such as the Mediterranean diet appear protective against cognitive decline (Heneka et al. 2025). Finally, socio‐economic disadvantage, through reduced access to dental and medical care, lower educational attainment and clustering of unhealthy behaviours, is linked both to a higher burden of periodontitis (WHO 2022) and to an increased risk of dementia (Mfene and Pillay 2024; Livingston et al. 2024). These factors converge on chronic low‐grade systemic inflammation, which may interact with genetic susceptibility and microbial dysbiosis to accelerate neurodegenerative processes.

## 6 Current Limitations in the Evidence

Despite a coherent and biologically plausible framework, the evidence linking periodontitis to ad remains predominantly associative, and several limitations warrant caution. Most human data derive from cross‐sectional and observational studies, which cannot establish causation. Shared risk factors, including advancing age, smoking, diabetes and socio‐economic disadvantage, may confound the observed associations, and reverse causality is a particular concern, because cognitive impairment itself contributes to deteriorating oral hygiene and self‐care. Longitudinal and interventional designs are therefore required before causal inferences can be drawn.

Methodological heterogeneity further complicates interpretation. Periodontitis is defined inconsistently across studies, with variation in clinical thresholds, indices and case definitions that hampers comparison and pooling of results. Similarly, ascertainment of dementia and ad ranges from registry codes and clinical diagnosis to biomarker‐confirmed cohorts, and misclassification is common. These inconsistencies contribute to the variable and at times conflicting prevalence and association estimates reported in the literature.

Mechanistic insights rely heavily on animal models and in vitro systems, which do not fully recapitulate the chronicity and complexity of human periodontitis or human neurodegeneration, limiting their translational relevance. The extent to which oral pathogens or their components translocate to, and persist within, the human brain remains uncertain, and some findings, including the detection of periodontal pathogens in brain tissue, await independent replication. Reports in this field have not been uniformly reproducible, and null or negative studies may be underrepresented.

Finally, whether periodontal treatment meaningfully alters cognitive trajectories has not been established. Although some observational cohorts and quasiexperimental studies suggest benefit, the largest randomised trial to date, a Phase 2/3 study of atuzaginstat, showed benefit confined to a *P. gingivalis* ‐positive subgroup rather than the overall cohort (Sabbagh and Decourt 2022). Available intervention data therefore remain heterogeneous and, on balance, inconclusive. The oral–brain axis should accordingly be regarded as a promising but still unproven hypothesis, and the present review should be read as a synthesis of converging mechanistic plausibility rather than of established causation.

## 7 Conclusion and Future Perspectives

Whereas existing reviews of the periodontitis–ad relationship have centred on microbial translocation and the gingipain hypothesis, the present review's distinct contribution is twofold: It reframes the OPG/RANKL system as a candidate bone–brain interface and advances the hypothesis that, because OPG acts as a decoy receptor for RANKL, elevated OPG in ad could limit the availability of neuroprotective RANKL. This review also identifies circulating endocan as a shared, treatment‐responsive vascular biomarker suited to longitudinal and interventional testing. Emerging evidence supports a multifactorial relationship between periodontitis and ad that also encompasses shared genetic (APOE ε4 and OPG), osteoimmune (OPG/RANK/RANKL) and vascular‐inflammatory mechanisms. A recurring theme across these domains is their convergence on microglial priming and sustained neuroinflammation, which the oral–brain axis frames as a route by which chronic periodontal inflammation may contribute to neurodegeneration, although current evidence remains largely associative and causality has not been established. Modifiable risk factors, including oral health, represent potential targets for early intervention. Future research should prioritise longitudinal and mechanistic studies to clarify causality, together with adequately powered randomised trials using cognitive and biomarker endpoints, to determine whether reducing periodontal and systemic inflammatory burden alters neurodegenerative outcomes. If confirmed, integrating oral healthcare into broader preventive strategies may offer a novel approach to mitigating the risk and progression of ad.

## Author Contributions

**Salma Kabbashi:** conceptualization, writing – original draft, writing – review and editing. **Mai Ahmed:** writing – original draft. **Manogari Chetty:** writing – review and editing.

## Funding

The authors declare that financial support was received for the research and/or publication of this article. Research reported in this study was supported by the South African Medical Research Council under a Self‐Initiated Research Grant. The views and opinions expressed are those of the authors and do not necessarily represent the official views of the SA‐MRC. Additional financial support for the research, authorship and/or publication of this article was provided by the UWC Research Chair for the SBDG project under the University Research Chairs Program. The work reported herein was made possible through funding from the South African Medical Research Council through its Division of Research Capacity Development under the SAMRC Research Capacity Development Initiative (RCDI) Programme. The content and findings reported/illustrated are the sole deduction, view and responsibilty of the researcher and do not reflect the official position and sentiments of tehe SAMRC.

## Conflicts of Interest

The authors declare no conflicts of interest.

## Data Availability

No data were used for the research described in the article.
